# A framework for flow time measured by Doppler ultrasound

**DOI:** 10.1186/s13089-025-00414-8

**Published:** 2025-01-21

**Authors:** Jon-Emile S. Kenny

**Affiliations:** 1 Health Sciences North Research Institute, Sudbury, ON Canada; 2 Flosonics Medical, Toronto, ON Canada

## Abstract

The duration of mechanical systole—also termed the flow time (FT) or left ventricular ejection time (LVET)—is measured by Doppler ultrasound and increasingly used as a stroke volume (SV) surrogate to guide patient care. Nevertheless, confusion exists as to the determinants of FT and a critical evaluation of this measure is needed. Using Doppler ultrasound of the left ventricular outflow tract velocity time integral (LVOT VTI) as well as strain and strain rate echocardiography as grounding principles, this brief commentary offers a model for the independent influences of FT. This framework establishes that systolic duration is directly proportional to the distance traversed by a single cardiac myocyte and indirectly proportional to its shortening velocity. Grossly, this translates to a direct relationship between FT and the LVOT VTI (i.e., SV) and an indirect relationship with mean ejection velocity. Thus, changes in the systolic time can infer SV change, so long as other cardiac parameters are considered.

Flow time (FT) is the duration of mechanical systole, usually measured in milliseconds (ms) [1, 2]. FT was, historically, obtained by analysis of the carotid pulse—assessed from the onset of the systolic upstroke to the trough of the incisural notch [1]. Fundamentally, FT is the time that the aortic valve is open and ejecting blood and is, accordingly, also known as left ventricular ejection time (LVET) (Fig. 1A) [1, 2]. Early studies related FT to stroke volume (SV) though more recent evaluations in the intensive care unit (ICU) considered FT to be a measure of preload [1–3]; nevertheless, debate about the true physiological determinants of FT led some prominent intensivists to declare that a ‘critical evaluation’ of FT is needed [4, 5].

Increasingly, Doppler ultrasound of the common carotid artery is used to measure FT (Fig. 1B) as surrogate for SV change (SV_∆_) and, in turn, to evaluate a patient for ‘fluid responsiveness’ (FR), when corrected for heart rate (HR) [6–10]. Given this newfound clinical application, a better physiological grounding of FT is needed. This brief commentary offers a framework for *time* as a metric of left ventricular function. More specifically, it is proposed that FT is directly related to SV, but inversely related to mean ejection velocity. Both Doppler ultrasound of the left ventricular outflow tract (LVOT) and stress and strain echocardiography are used as conceptual models to connect the duration of mechanical systole (i.e., FT and LVET) to SV, contractility, afterload and HR.

**Fig. 1:**
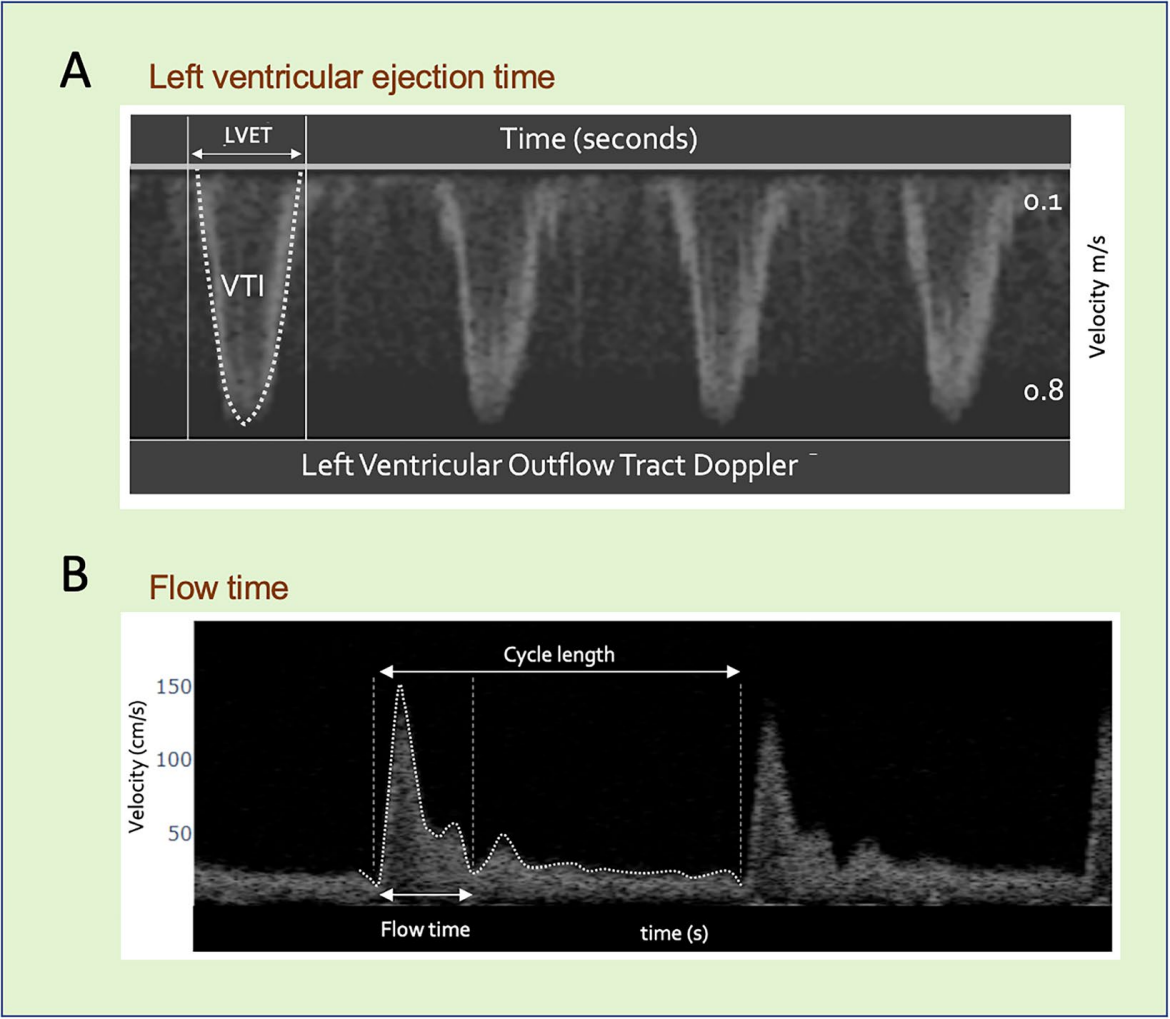
The left ventricular ejection and flow times. **A**) 4 cardiac cycles obtained via trans-esophageal echocardiography. Velocity increases in the downwards y-axis and x-axis is time. LVET is the duration the aortic valve is open and ejecting blood, the left ventricular ejection time. VTI is velocity time integral. **B**) Flow time from the common carotid artery. Velocity increases upwards on the y-axis and the x-axis is time

## Time and the left ventricular outflow tract

**Fig. 2:**
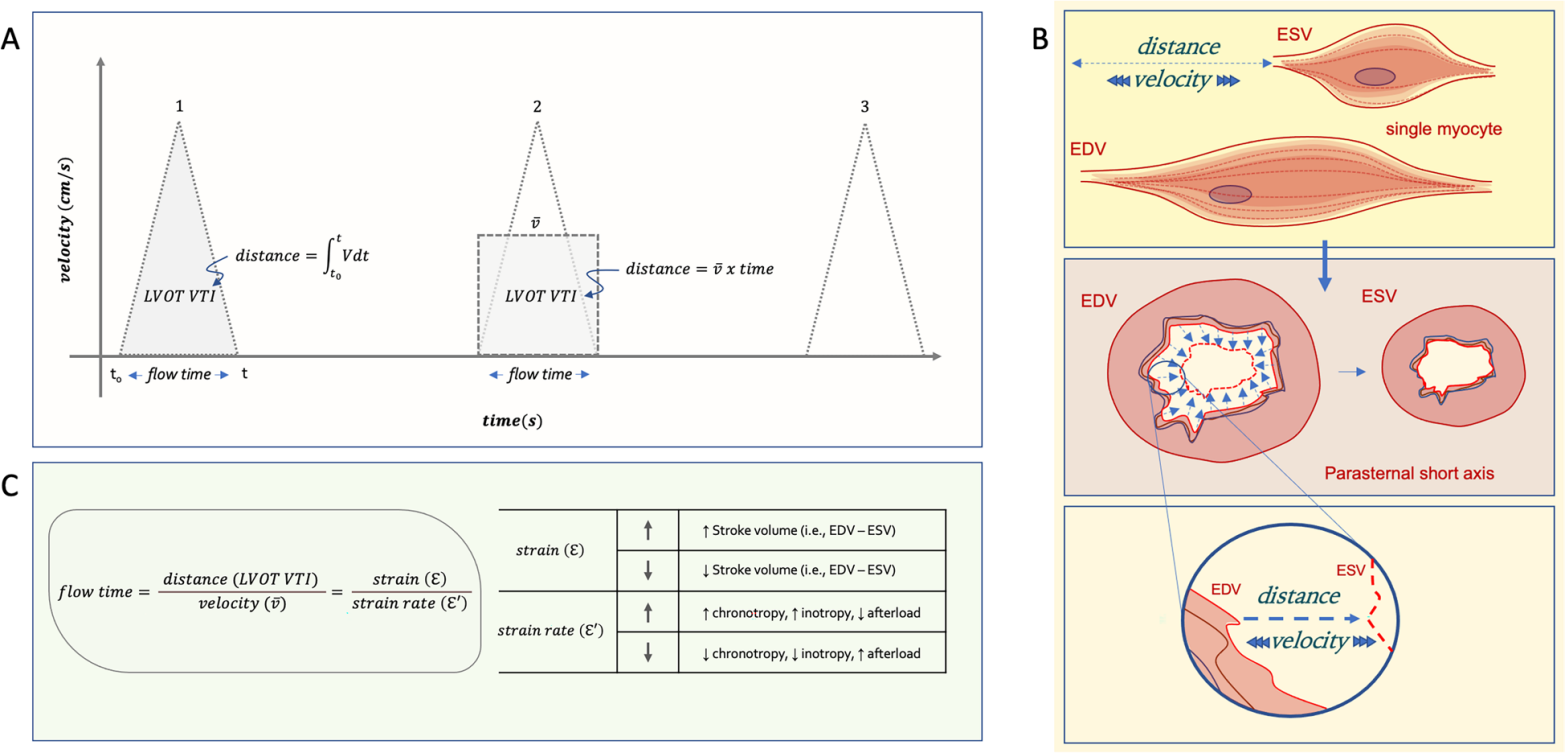
Relating left ventricular outflow tract Doppler ultrasound and strain echocardiography to flow time. **A**) cartoon of 3 cardiac cycles with equal left ventricular outflow tract velocity time integral (LVOT VTI); see text for details. **B**) Analogy using single cardiac myocyte to understand the relationship between time, distance and velocity. **C**) Framework relating flow time to LVOT VTI (i.e., distance), mean ejection velocity ($\overline{v }$), strain (ℇ) and strain rate (ℇ'). EDV is end-diastolic volume, ESV is end-systolic volume. Note that increasing preload (i.e., EDV) will also increase flow time, but only if EDV rises relative to ESV (i.e., increased stroke volume) and with constant mean ejection velocity

Doppler ultrasound of the LVOT generates a roughly triangular-shaped spectrogram with velocity (i.e., centimeters per second, cm/s) on the y-axis and time (i.e., seconds) on the x-axis Fig. 2A) [11]. From this Doppler envelope, the distance that the blood travels from the LVOT is calculated, in centimeters (cm), by integrating the velocity–time curve as follows:

1 $$distance = \int\limits_{{t_{0} }}^{t} {v \ dt}$$

where t_0_ and t are the onset and offset of mechanical systole (i.e., FT or LVET), respectively, and v is the instantaneous velocity at any given time throughout systole. A mathematically equivalent way of expressing this is to use the mean ejection velocity ($\overline{v }$) during mechanical systole, as follows:

2 $$distance = \overline{v} \times time$$

From Eqs. 1 and 2 the ‘distance’ that is calculated is also called the LVOT velocity time integral (VTI) because it is the area under the velocity–time curve (Fig. 2A). If we multiply the LVOT VTI (i.e., distance) by the cross-sectional area (CSA) of the LVOT, SV is obtained in cm^3^ or milliliters. Clinically, the CSA of the LVOT is often assumed to be constant; thus, LVOT VTI (i.e., distance) change is directly related to SV_∆_. By rearranging Eq. 2, we see how *time* relates to LVOT VTI.

3 $$time = \frac{ LVOT \ VTI }{{\overline{v}}}$$

Per Eq. 3, the duration of systole is directly proportional to LVOT VTI, but indirectly related to $\overline{v }$. Therefore, increased FT could mean LVOT VTI (i.e., SV) augmentation and/or decreased $\overline{v }$ and vice versa. Nevertheless, Eq. 3 might be perplexing given the conceptual and physiological linkage between the numerator (i.e., LVOT VTI) and the denominator (i.e., mean ejection velocity). To address this, an analogy using a single cardiac myocyte is proposed (Fig. 2B). The time it takes for a single myocyte to contract is directly proportional to the distance, or length, the myocyte shortens (i.e., extent of deformation) and indirectly proportional to its shortening velocity (i.e., rate of deformation). To tease out how cardiac loading affects the extent and rate of deformation, independently, strain and strain rate echocardiography are explored.

## Strain and strain rate echocardiography

Abraham and colleagues studied strips of heart muscle and found a strong, linear correlation between the change in myocyte length and myocardial strain (ε) [12]. Additionally, they found that strain rate (ε′) directly and indirectly correlated with contractility and afterload, respectively. Carrying forward the mathematical relationship, described above, we arrive at:

4 $$time = \frac{ distance }{{velocity }} = \frac{ extent\, of \,myocyte \,deformation }{{rate\, of \,myocyte\, deformatiom }} = \frac{ strain (\varepsilon ) }{{strain \, rate (\varepsilon^{\prime}) }}$$

Though ε and ε′ echocardiography are regional measures, when extrapolated to global cardiac function ε (i.e., the extent of deformation) relates to SV while ε′ (i.e., the rate of deformation) associates with contractility and afterload (Fig. 2C). Indeed, animal models have confirmed that ε tracks SV_∆_ well while ε′ is directly related to contractility and indirectly related to afterload [13–16]. More complicated, however, is the effect of preload on ε and ε′. Both ε and SV are enhanced by preload (i.e., by increasing end-diastolic volume, EDV, relative to the end-systolic volume, ESV) [13, 14, 17]; however, the effect of preload on ε′ is more nuanced. When single cardiac myocytes are studied, increasing preload does not increase shortening velocity (i.e., ε′) [18]; nevertheless, in vivo, the effect on ε′ is also tied to how preload modulates afterload (i.e., does the rise in SV also change arterial elastance), as described by Burns and colleagues [19]. This interdependence of cardiac loading parameters confounds time as a measure of LV function but also ties together seemingly disparate findings. For instance, increased afterload might prolong FT by selectively reducing shortening velocity (i.e., decreasing the denominator of Eqs. 3 or 4) [20]; conversely, if elevated afterload truncates SV to a greater extent (i.e., by raising ESV, shrinking the numerator of Eqs. 3 or 4), then the FT will fall in response to increased afterload [21]. On the other hand, to the extent that decreased afterload raises SV, FT increases [22]; however, if diminished afterload concurrently augments deformation rate (ε′) to a greater extent, then systolic time falls (e.g., when severe aortic stenosis is corrected) [23].

## Heart rate correction

Thus far, FT was discussed without any heart rate (HR) correction, which is commonly performed clinically. There are numerous equations used to correct for heart rate (e.g., Wodey, Bazett, Weissler) [24], but why might this be physiologically necessary? If the truncation of systole with increased HR is due only to reduced LV filling (i.e., EDV or preload), then the fall in absolute FT would directly reflect decreased ε (or SV, globally). However, the chronotropic response also increases myocyte shortening velocity—the so-called ‘Bowditch effect [18]’—which diminishes systolic time for any given ε. Accordingly, there is a mild-to-moderate correlation between HR and ε′ [25, 26]; correcting for HR, in theory, accounts for this phenomenon. Beyond accounting for chronotropy, there are no known equations that adjust for inotropic or afterload state when correcting systolic time.

## Clinical implications

Decreasing FT (i.e., LVET) over time is a known, independent predictor of incident congestive heart failure (CHF) [27]. Furthermore, FT has been used to monitor inpatient and outpatient therapy for patients with reduced ejection fraction and CHF [28]; this population has significantly reduced FT [28, 29] which negates the notion that FT is a marker of preload because these patients have increased left ventricular end-diastolic volume (i.e., preload) despite substantially reduced FT. Based on the model put forth above, the low FT is most likely due to reduced SV (i.e., from high ESV). Importantly, both positive inotropes and vasodilators augment FT in these patients [22, 28]. Because both of these classes of agents increase ε′ (i.e., reduce FT per the model above), the observed rise in FT must mean these agents significantly increase the extent of LV shortening (i.e., the SV) by decreasing ESV. Nevertheless, within the realm of CHF, a prolonged FT is does not necessarily imply optimal cardiac function. For instance, increased systemic vascular resistance and LV wall thickness both decrease ε′ [16, 25], which prolongs FT. This could explain an observed U-shape curve between FT and all-cause mortality in patients with coronary artery disease [30].

Additionally, changes in corrected flow time of the carotid artery have been successfully related to SV_∆_ in critically-ill patients receiving a preload challenge either by passive leg raising or intravenous crystalloids [6–8]. Per Eqs. 3 and 4, an increase in corrected FT reflects increased SV only when afterload and contractility (i.e., ε′ or $\overline{v }$) remain constant. This is probably a fair assumption when preload is administered, though increased SV can reduce afterload in septic patients [31]. If reduced arterial load were to simultaneously increase ε′, then the rise in FT with SV would be blunted. Interestingly, Barjaktarevic and colleagues found a lower sensitivity than specificity [6]; increased false negatives could be a consequence of increased ejection velocity.

An important caveat for the aforementioned is the assumption that LVET (i.e., measured at the aortic valve) is equivalent to the FT measured in a large central artery like the common carotid. While the time of mechanical systole measured at the common carotid is strongly correlated with the time that the aortic valve is open and ejecting blood [23], the relationship between aortic valve opening and the duration of systole in distal, smaller arteries may not be as direct. More specifically, measuring arterial blood velocity closer to the arterioles—the main source of wave reflections—reveals earlier systolic deceleration and velocity reversal at the dicrotic notch [32]. This could occur while the aortic valve is still open, meaning that FT in a distal artery might underestimate absolute LVET. A similar phenomenon is possible in the common carotid artery following catastrophic brain injury where cerebral vascular resistance is significantly increased, enhancing early wave reflections. Nevertheless, measuring FT *change* in a distal artery before and after a hemodynamic intervention (i.e., a dynamic paradigm) might still track changes in LVET but this is not known.

## Conclusion

Systolic duration measured by Doppler ultrasound is directly proportional to the distance traversed by a single cardiac myocyte and indirectly proportional to the velocity of its shortening. Globally, this translates to a direct relationship between time and the LVOT VTI (or SV) and an indirect relationship with mean ejection velocity. Studies of myocardial strain and strain rate clarify this relationship. Increased contractility, chronotropy and decreased afterload all increase ε′ which reduces FT and vice versa. Changes in the systolic time domain can be used to infer SV_∆_, so long as other cardiac parameters are considered.

## Data Availability

Not applicable.

## Abbreviations

FT: Flow time
ms: Milliseconds
LVET: Left ventricular ejection time
SV: Stroke volume
ICU: Intensive care unit
SV_∆_: Stroke volume change
FR: Fluid responsiveness
HR: Heart rate
LVOT: Left ventricular outflow tract
cm: Centimeters
$\overline{v }$: Mean ejection velocity
VTI: Velocity time integral
CSA: Cross sectional area
ε: Myocardial strain
ε′: Myocardial strain rate
ESV: End-systolic volume
EDV: End-diastolic volume
CHF: Congestive heart failure
